# Gβ1γ2 activates phospholipase A_2_-dependent Golgi membrane tubule formation

**DOI:** 10.3389/fcell.2014.00004

**Published:** 2014-02-28

**Authors:** Marie E. Bechler, William J. Brown

**Affiliations:** Department of Molecular Biology and Genetics, Cornell UniversityIthaca, NY, USA

**Keywords:** Golgi complex, membrane tubules, phospholipids, Gβγ, heterotrimeric G proteins, PLA_2_

## Abstract

Heterotrimeric G proteins transduce the ligand binding of transmembrane G protein coupled receptors into a variety of intracellular signaling pathways. Recently, heterotrimeric Gβγ subunit signaling at the Golgi complex has been shown to regulate the formation of vesicular transport carriers that deliver cargo from the Golgi to the plasma membrane. In addition to vesicles, membrane tubules have also been shown to mediate export from the Golgi complex, which requires the activity of cytoplasmic phospholipase A_2_ (PLA_2_) enzyme activity. Through the use of an *in vitro* reconstitution assay with isolated Golgi complexes, we provide evidence that Gβ1γ2 signaling also stimulates Golgi membrane tubule formation. In addition, we show that an inhibitor of Gβγ activation of PLA_2_ enzymes inhibits *in vitro* Golgi membrane tubule formation. Additionally, purified Gβγ protein stimulates membrane tubules in the presence of low (sub-threshold) cytosol concentrations. Importantly, this Gβγ stimulation of Golgi membrane tubule formation was inhibited by treatment with the PLA_2_ antagonist ONO-RS-082. These studies indicate that Gβ1γ2 signaling activates PLA_2_ enzymes required for Golgi membrane tubule formation, thus establishing a new layer of regulation for this process.

## Introduction

The formation of transport carriers from the mammalian Golgi complex requires regulation for precise spatial and temporal trafficking within a cell (Yang et al., 2011). Cargo transport from the Golgi complex involves both membrane bound vesicles and membrane tubules. Cargo exiting the *trans* Golgi network (TGN), such as ts045 VSV-G, as well as retrograde cargo from the cis Golgi to the endoplasmic reticulum, has been visualized to travel in long, 60–80 nm diameter membrane-bound tubule carriers (Bechler et al., 2012; Ha et al., 2012; Martinez-Alonso et al., 2013). Additionally, vesicle markers have been shown to partially colocalize with these membrane tubules, from which vesicles may bud (Puertollano et al., 2001, 2003; Waguri et al., 2003). The outward budding of a nascent vesicle initially requires positive membrane curvature that is also necessary for forming membrane tubule carriers. However, little is known about the mechanisms that regulate the positive curvature needed to form both vesicles and membrane tubules from the Golgi complex (Bechler et al., 2012; Ha et al., 2012).

The membrane-bending capability of phospholipid-modifying enzymes may provide a mechanism for forming the initial curvature of a vesicle or of a membrane tubule. Indeed, a series of pharmacological studies using phospholipase A_2_ (PLA_2_) antagonists indicate that phospholipases are important for the formation of membrane tubules (de Figueiredo et al., 1998, 1999, 2000; Drecktrah and Brown, 1999; Polizotto et al., 1999). Recent studies have since identified specific phospholipase (PLA) enzymes that contribute to different levels of Golgi membrane tubule formation: cPLA_2_α (San Pietro et al., 2009), PLA2G6-A (Ben-Tekaya et al., 2010), and platelet activating factor acetylhydrolase Ib (PAFAH Ib) (Bechler et al., 2010). These phospholipases have partially overlapping function and contribute to distinct aspects of Golgi structure and trafficking. However, it is not known how these PLA enzymes are individually regulated, nor is it understood how membrane tubules in general are regulated (Bechler et al., 2012; Ha et al., 2012).

The large, heterotrimeric G protein family, composed of various combinations of Gα, Gβ, and Gγ subunit isoforms, has been implicated in the regulation of Golgi trafficking, architecture, and vesicle budding (Stow et al., 1991; Denker et al., 1996; Jamora et al., 1997, 1999; Diaz Añel and Malhotra, 2005; Irannejad and Wedegaertner, 2010). Early studies with ilimaquinone (IQ), a metabolite of marine sponges that vesiculates the Golgi complex, revealed a role for Gαs and Gαi-3 as well as Gβγ subunits at the Golgi (Takizawa et al., 1993; Jamora et al., 1997, 1999). Gαi-3 and Gαq have also been implicated in the control of Golgi architecture and trafficking (Denker et al., 1996). Specific Gβγ subunits, Gβ1γ2 and Gβ2γ3, upon overexpression in mammalian cells, dramatically affect Golgi membrane structure and alter trafficking, likely through activation of PKCη and PKD at the TGN (Jamora et al., 1999; Diaz Añel and Malhotra, 2005). Additionally, studies suggest that particular Gβγ isoforms are capable, upon GPCR stimulation, of translocating from the plasma membrane to the Golgi complex, where the Gβγ may trigger vesiculation of the Golgi complex as well as increased transport (Akgoz et al., 2006; Saini et al., 2010). The localization of Gβ1γ2 specifically to the Golgi complex results in fragmentation of the Golgi and TGN, and inactivation of endogenous Gβγ significantly affects secretory trafficking (Irannejad and Wedegaertner, 2010).

In addition to roles in stimulating vesiculation, Gβγ has been implicated in regulating brefeldin A (BFA)-stimulated membrane tubules. For example, Golgi and endosome membrane tubules stimulated by BFA were inhibited by the biscolaurine alkaloid isotetrandrine (ITD) (Chan et al., 2004), an inhibitor of Gβγ-mediated PLA_2_ enzyme activation (Hashizume et al., 1991; Akiba et al., 1992, 1995). This membrane tubule inhibition by ITD is believed to be an effect of preventing activation of PLA_2_ enzymes through yet unknown Gβγ subunits. Put together, it is likely that Gβγ simultaneously activates pathways that promote PLA_2_ enzyme activity to generate positive curvature (for both membrane tubules and vesicle formation) as well as activate machinery for vesicle fission.

Reported here is the use of a cell free reconstitution assay (Cluett et al., 1993; Banta et al., 1995) to further test the idea that a Gβγ signaling pathway can activate PLA_2_-dependent membrane tubule formation. This method permits direct examination of individual components for contributions to Golgi membrane tubule formation.

## Methods and materials

### Reagents

Sprague-Dawley male rats were obtained from Charles River Breeding Laboratories, Inc. Isotetrandrine (ITD) and ONO-RS-082 (ONO) were purchased from BioMol Research Laboratories, Inc. Recombinant Gβ1 and γ2 were prepared as described (in buffer containing 150 mM NaCl, 1mM EDTA, 1 mM DTT, 0.1% (w/v) Chaps, and 20 mM Tris, pH 8.0) (Mayeenuddin et al., 2006) and was kindly provided by Dr. James Garrison (University of Virginia, Charlottesville, VA).

#### In vitro Golgi tubulation assays

**Figure 1:**
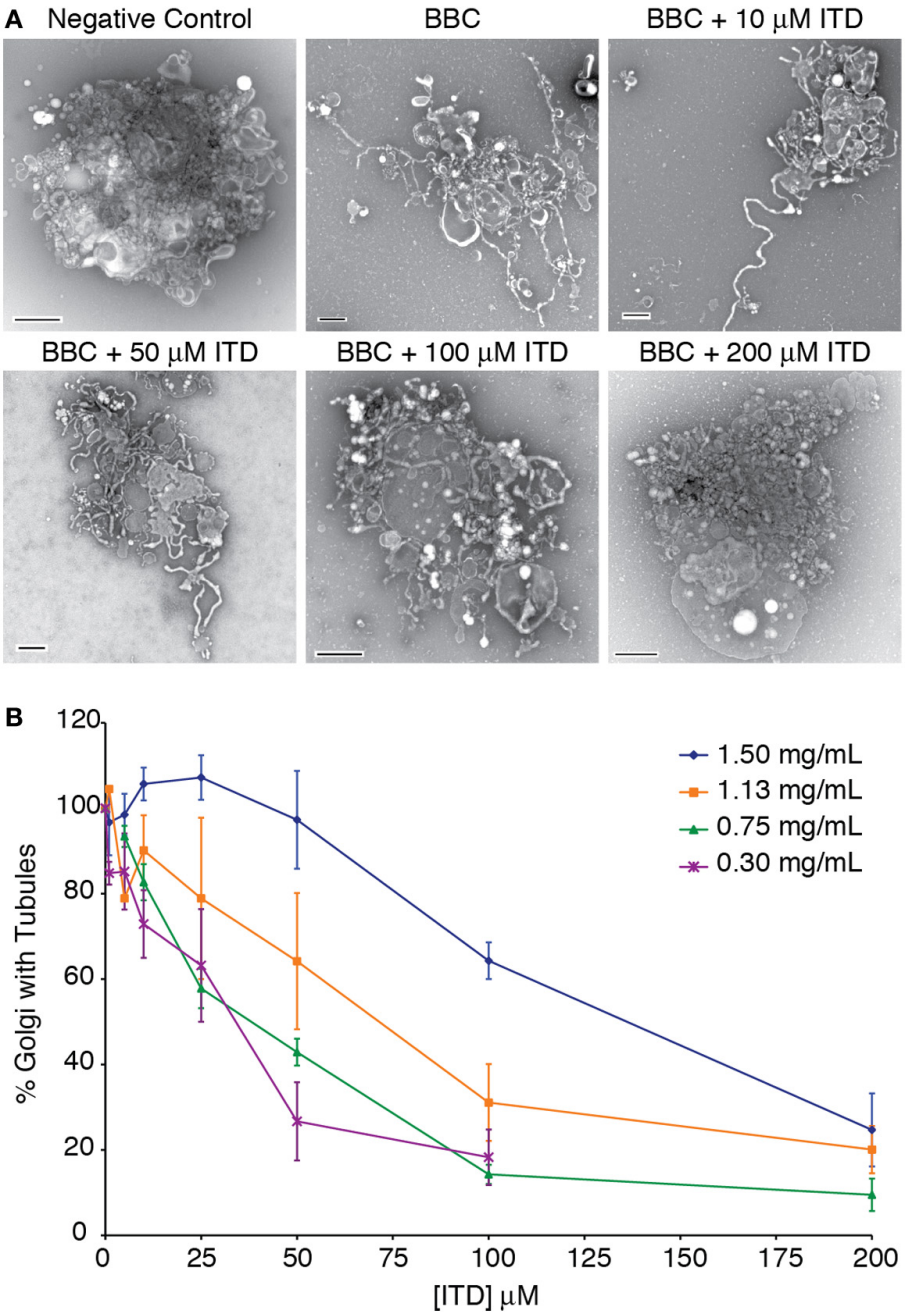
**Dose-dependent inhibition of cytosol-stimulated Golgi membrane tubules. (A)** Example negative stain electron micrographs of tubulated and non-tubulated Golgi from the *in vitro* reconstitution assay. Bovine brain cytosol (BBC, 1.5 mg/ml) was incubated with the indicated concentration of ITD and added to isolated Golgi complexes. Control Golgi complexes were incubated with 0.2 mg/ml BSA. Scale bars = 500 nm. **(B)** Quantification of the percent of Golgi complexes with membrane tubules, normalized to the maximum percent of Golgi with tubules in the presence of each BBC concentration shown. Averages are shown from minimum of three replicates, error bars = s.e.m.

Analysis of Golgi membrane tubule formation was achieved through the use of a well-characterized *in vitro* reconstitution system developed in our laboratory (Banta et al., 1995; de Figueiredo et al., 2000), with several important modifications as described below. Intact Golgi complexes from rat liver were enriched following procedures as described (Cluett et al., 1993; Banta et al., 1995). Bovine brain cytosol (BBC) was prepared and an *in vitro* Golgi tubulation assay was performed as described (Banta et al., 1995). Briefly, all frozen reagents were rapidly thawed at 37°C and then kept on ice until use. Reaction mixtures containing BBC, purified protein, ± inhibitors mixed with tubulation assay buffer (50 mM KCl, 1 mM MgCl_2_, 25 mM Tris, 10 mM HEPES, pH 7.4) and ATP (final concentration, 50 μM), were prepared as indicted in the results. For some experiments bovine serum albumin (BSA) was used as a negative control, in that it did not stimulate or inhibit membrane tubule formation, as previous shown (de Figueiredo et al., 2000; Bechler et al., 2010). Other studies also demonstrated that non-specific phospholipid hydrolysis by snake venom PLA_2_ did not induced tubule formation (Bechler et al., 2010). Golgi aliquots and reaction mixtures were pre-warmed to 37°C for 15 min, after which the reaction mix was gently mixed 1:1 into the Golgi aliquots, and then incubated for 15 min at 37°C. Samples were spotted onto Formvar- and carbon-coated EM grids, followed by staining with 2% phosphotungstic acid, pH 7.2. Grids were viewed on a FEI Morgagni 268 transmission electron microscope. Grids were coded and counted blind. Golgi complexes on negative stain grids were identified by characteristic morphology (Banta et al., 1995; de Figueiredo et al., 1999; Polizotto et al., 1999), with a minimum diameter of 1 μm. Previous studies using immunogold labeling of Golgi-localized α-mannosidase II on whole mount preparations determined that reproducibly ~50% of the negatively stained profiles are intact Golgi complexes (de Figueiredo et al., 1999). Golgi phenotypes were counted based on morphology. Golgi complexes scored as not tubulated had a characteristic appearance of tightly interwoven sac, vesicular and tubular membranes. Golgi complexes scored as tubulated were distinguished as having at least one 60–80 nm diameter tubule extension of more than 1 μm or having multiple 60–80 nm tubule extensions of at least 500 nm in length. It is worth noting that this assay does not precisely measure tubule number and length because of the difficulty of accurately determining these features in a complicated negatively stained Golgi complex, which contains membrane tubules that weave over and under the stack proper (see for example, Figure 1). Therefore, any changes due to inhibitory or stimulatory factors examined here will likely be under-estimated. At least 70 Golgi profiles per condition were counted for each experiment, with a minimum of three experiments. Either One-Way ANOVA or two-tailed *t*-tests assuming unequal variance were used to analyze significance of the data, as indicated in figure legends.

## Results

A preparation of bovine brain cytosol (BBC) stimulates Golgi membrane tubule formation in an *in vitro* reconstitution assay (Cluett et al., 1993; Banta et al., 1995), which can be inhibited by PLA_2_ inhibitors (de Figueiredo et al., 1999) and stimulated by the addition of purified PAFAH Ib (Bechler et al., 2010, 2012; Ha et al., 2012). Here we describe the use of this *in vitro* reconstitution assay to assess the contribution of Gβ1γ2 and subsequent stimulation of PLA_2_ enzymes to the formation of Golgi membrane tubules.

### Isotetrandrine inhibits cytosol-stimulated Golgi membrane tubules *in vitro*

Previous studies have shown that ITD inhibits BFA-stimulated Golgi membrane tubules in mammalian cells (Chan et al., 2004). To determine if ITD similarly inhibits cytosol-stimulated Golgi tubules *in vitro*, we tested a range of ITD concentrations with varying BBC concentrations. ITD exhibited a dose-dependent inhibition of BBC-stimulated membrane tubules and showed a positive correlation between concentration of cytosol and the IC_50_ for membrane tubule inhibition (Figure 1). This IC_50_ of membrane tubule inhibition is dependent on the individual preparation of BBC, as there is natural variation between different preparations of BBC. We found an IC_50_ range from 25 to 100 μM with cytosol concentrations that achieve maximum number of tubulated Golgi membranes. Therefore, for each BBC preparation, the appropriate IC_50_ was determined before further experimentation.

### Isotetrandrine inhibits Golgi-associated components

**Figure 2:**
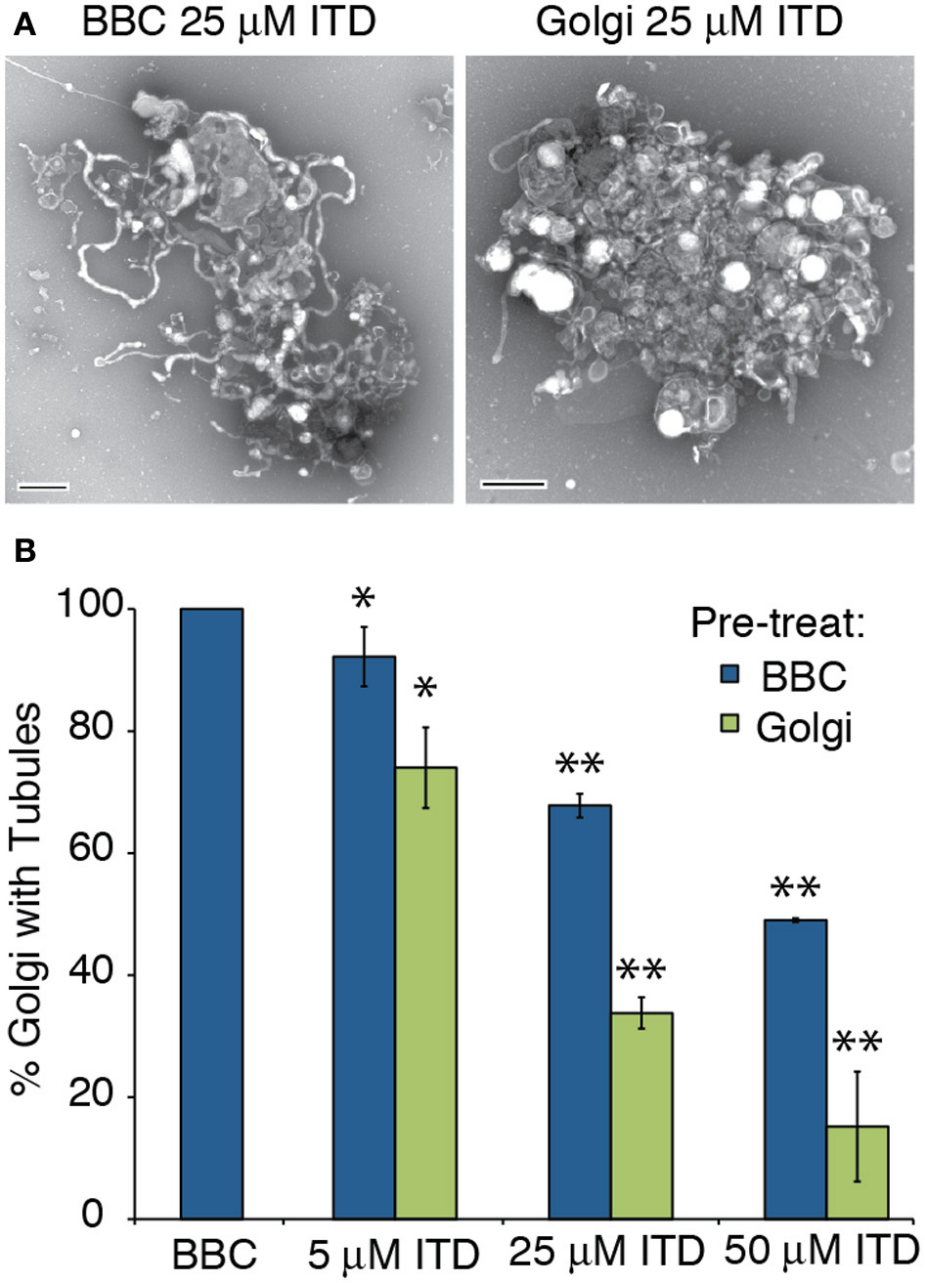
**ITD inhibits Golgi-membrane associated proteins**. Various concentrations of ITD were either pre-incubated at 37°C for 15 min with BBC or Golgi membranes before combining the cytosol and Golgi, followed by further incubation at 37°C for 15 min. (**A**) Representative negative stain Golgi treated with ITD, which was pre-incubated as labeled. Scale bars = 500 nm. (**B**) Quantification of the percent of Golgi with membrane tubules, normalized to BBC alone (1.5 mg/ml). ITD concentrations shown are the final concentration after mixing the Golgi and cytosol. Error bars = s.e.m. Differences between samples with ITD pre-treated BBC or Golgi were statistically different, with *p*-values < 0.04 (^*^) or 0.006 (^**^), determined using a two-tailed *t*-test for each ITD concentration.

The exact target of ITD is unknown (Hashizume et al., 1991; Akiba et al., 1992, 1995), therefore we tested whether the target of ITD was cytosolic or membrane-associated. The extent of Golgi membrane tubule inhibition was compared between ITD addition to the Golgi membranes themselves—presumably inhibiting a protein directly associated with the membranes—vs. ITD addition to the cytosol, inhibiting a cytosolic target. Either the BBC or Golgi membranes were pretreated with isotetrandrine, and then combined. ITD was more efficacious when Golgi membranes were pretreated, consistent with the idea that ITD inhibits a Golgi-associated target (Figure 2).

### Gβ1γ2 rescues ITD inhibition

**Figure 3:**
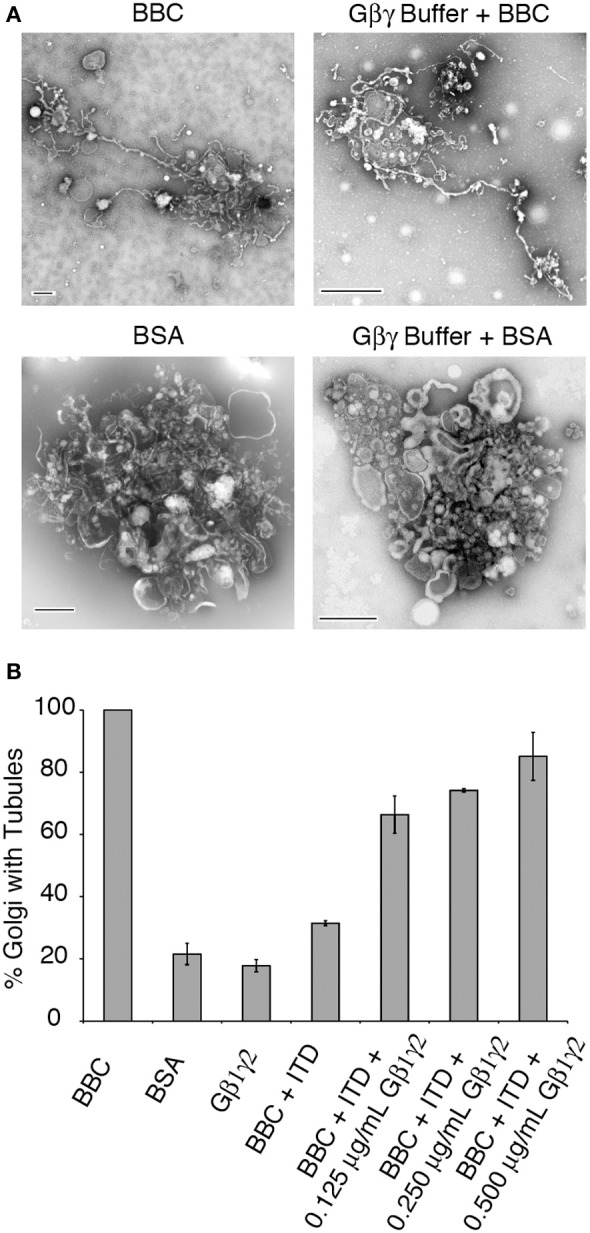
**Gβ1γ2 rescues ITD inhibition of cytosol-stimulated Golgi membrane tubules. (A)** Representative EM micrographs of Golgi treated with BBC (1.5 mg/ml) or BSA (0.2 mg/ml) in the presence or absence of the Gβγ buffer that contains CHAPS detergent. Membrane tubules and Golgi morphology were unaffected by the presence of the buffer. **(B)** Quantification of the fraction of Golgi with tubules, relative to the maximum percent of Golgi with tubules seen with BBC (1.5 mg/ml) alone. Final concentrations (after mixing the pre-incubated Golgi and cytosol) are shown for purified Gβγ. ITD final concentration was 25 μM. Error bars = s.e.m. Samples containing BBC + ITD+ Gβ1γ2 were statistically different with p-values < 0.003 compared to BSA, Gβ1γ2 alone, or BBC + ITD, as determined using ANOVA.

The pharmacological effect of ITD is to inhibit Gβγ activation of PLA_2_ enzymes (Hashizume et al., 1991; Akiba et al., 1992, 1995). To determine if ITD inhibits a pathway stimulated by Gβγ, as suggested by the previous pharmacological studies, Gβ1γ2 was added to the ITD-treated tubulation mixture and tested for its ability to stimulate Golgi membrane tubules. Gγ is prenylated on its C-terminus and must be kept soluble in low concentrations of the detergent CHAPS. We first tested whether the CHAPS-containing Gβγ buffer affects Golgi membranes or tubule formation. We found no effect on the percent of tubulated Golgi for either BBC-stimulated or BSA control treated Golgi complexes (Figure 3A). Of the various Gβγ isoforms, Gβ1γ2 was selected because it is the most abundant isoform in bovine brain and has been previously shown to affect Golgi architecture and trafficking (Diaz Añel and Malhotra, 2005; Irannejad and Wedegaertner, 2010). BBC-stimulated Golgi tubulation was inhibited to near-background levels by ITD (Figure 3B). Addition of increasing amounts of purified Gβ1γ2 protein to Golgi membranes treated with 25 μM ITD resulted in nearly complete restoration of membrane tubule formation to control levels (Figure 3B).

### Gβ1γ2 stimulates Golgi membrane tubule formation

**Figure 4:**
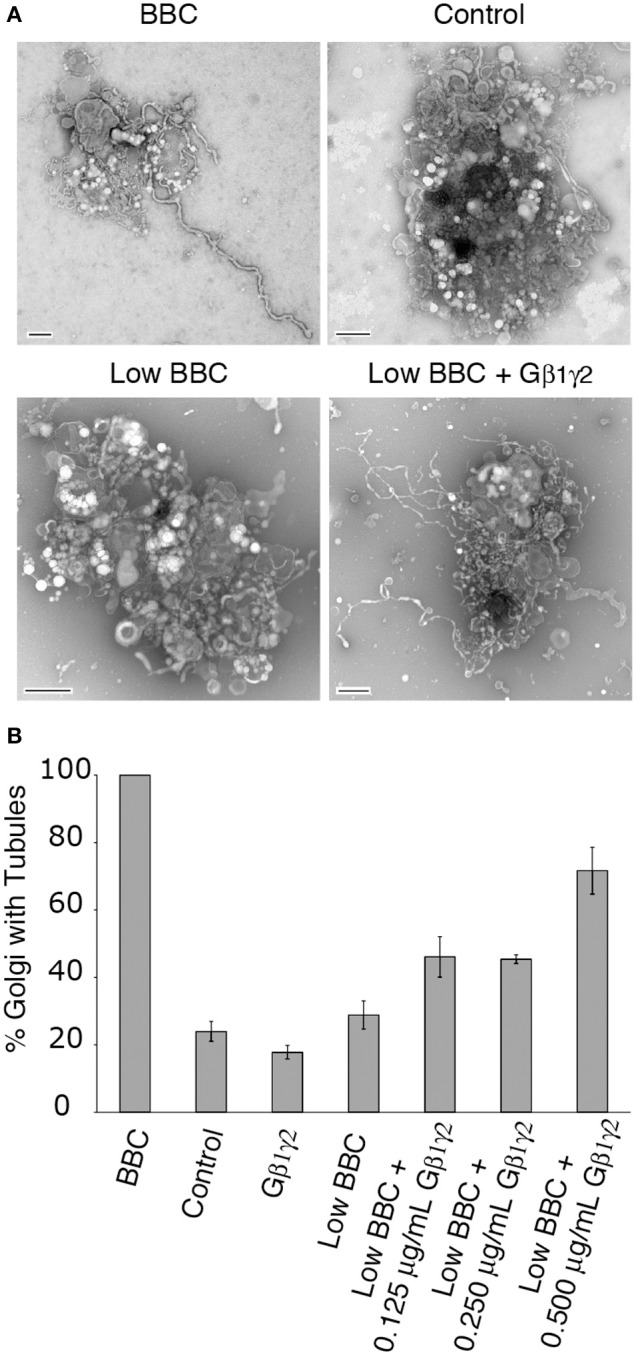
**Gβ1γ2 stimulates cytosol-dependent Golgi membrane tubulation. (A)** Representative EM micrographs of Golgi treated with BBC (1.5 mg/ml), a BSA control (0.2 mg/ml), Low BBC (0.15 mg/ml), or Low BBC + Gβ1γ2 (0.5 μg/ml Gβγ). Each condition was in the presence of the Gβγ buffer. Scale bar = 500 nm. (**B**) Quantification of the percent of Golgi with membrane tubules, normalized to the maximum amount of tubulated Golgi with BBC (1.5 mg/ml). Gβ1γ2 purified protein alone does not stimulate Golgi membrane tubules above background levels (no BBC control). In the presence of low cytosol concentrations (Low BBC 0.15 mg/ml), the addition of Gβ1γ2 stimulates membrane tubules. Error bars = s.e.m. Low BBC + 0.0125 μg/mL Gβ1γ2 was statistically different than samples of Control, Gβ1γ2 alone, or Low BBC, with *p*-values < 0.009 determined using ANOVA.

ITD inhibition of cytosol-stimulated Golgi tubules could be rescued by addition of purified Gβ1γ2, therefore we wanted to address whether Gβγ itself could stimulate membrane tubules in the reconstitution assay. Gβγ, when added alone to Golgi membranes, was insufficient to stimulate membrane tubules above background (Figure 4). Either Gβ1γ2 does not stimulate membrane tubules or a cytosolic component (e.g., a PLA_2_) is required to induce membrane tubule formation. Near background levels of membrane tubule formation are seen with low concentrations of BBC (sub-threshold levels), which can be used in combination with other factors that promote membrane tubule formation to achieve maximum Golgi membrane tubules (Polizotto et al., 1999). Therefore we tested whether Gβγ signaling requires a cytosol component by adding Gβγ to sub-threshold cytosol levels. Indeed, we found that the addition of Gβ1γ2 in increasing amounts stimulated the formation of membrane tubules in the presence of low BBC (Figure 4).

### Gβ1γ2-stimulated Golgi membrane tubules are PLA_2_ dependent

**Figure 5:**
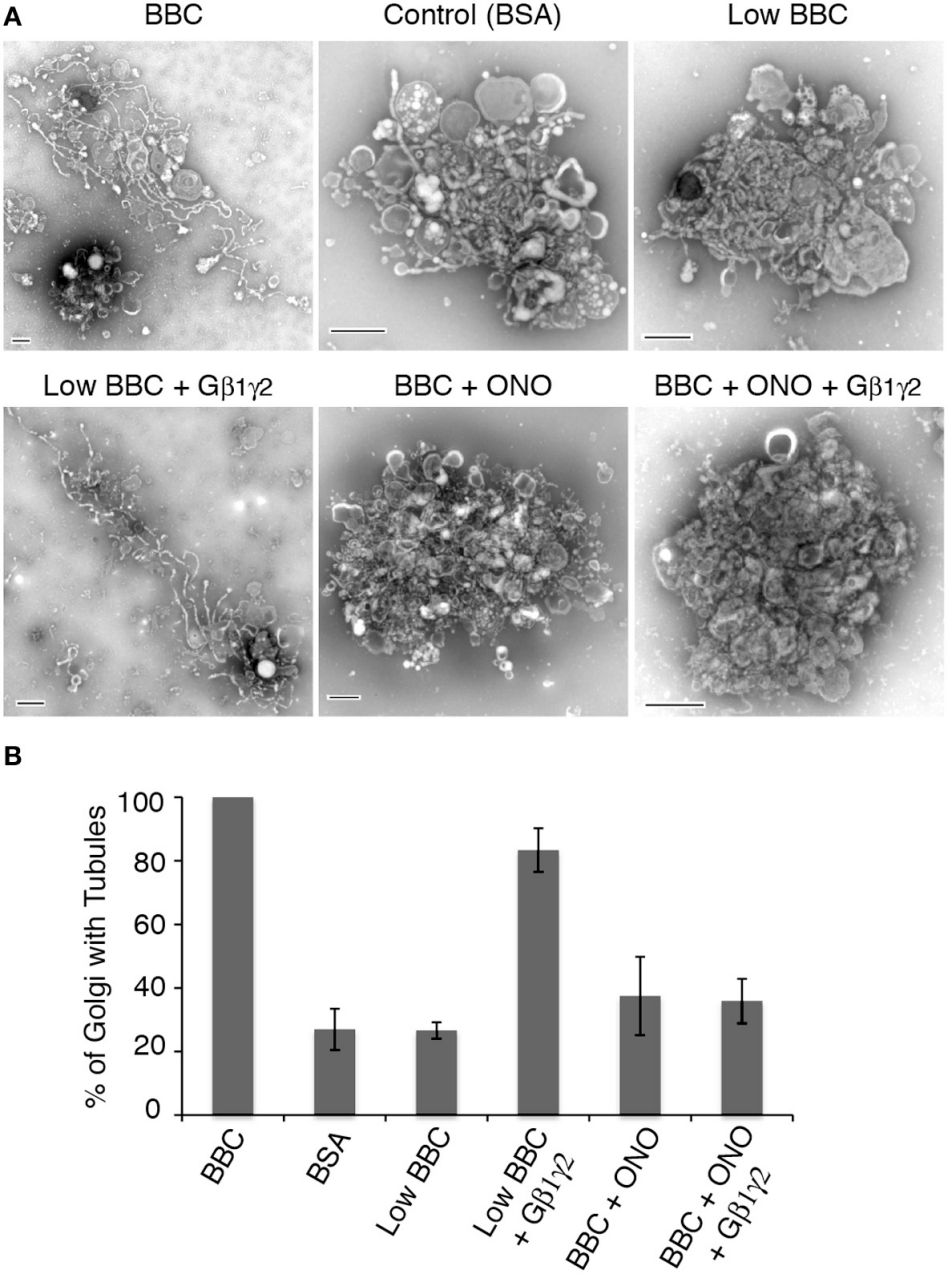
**Gβ1γ2 stimulation of Golgi membrane tubules is PLA_2_ activity dependent**. BBC (1.5 mg/ml) was pre-incubated with the PLA_2_ inhibitor ONO (at final concentration of 25 μM) at 37°C for 15 min, followed by the addition of Gβ1γ2 purified protein (final concentration of 0.4 μg/ml) to BBC before combining the cytosol and Golgi, and additional incubation at 37°C for 15 min. Low BBC was 0.15 mg/ml and the BSA, as a negative control, was at a final 1 mg/ml concentration. (**A**) Representative negative stain Golgi treated with ONO and Gβ1γ2 as labeled. Scale bars = 500 nm. (**B**) Quantification of the percent of Golgi with membrane tubules, normalized to BBC alone (1.5 mg/ml). Error bars = s.e.m. Golgi samples with ONO-treated BBC, BSA, or Low BBC were statistically different from Low BBC + Gβ1γ2 with *p*-values < 0.013, as determined using ANOVA.

The above studies show that BBC contains a component that is stimulated by Gβ1γ2, likely a protein downstream of the target of ITD. Previous studies have demonstrated that BBC-stimulated Golgi membrane tubules require PLA_2_ enzyme activity (Brown et al., 2003; Bechler et al., 2012), and ITD is suggested to inhibit a Gβ1γ2 activation of PLA_2_ enzymes (Akiba et al., 1995), so we tested whether Gβ1γ2-stimulation of membrane tubules also requires PLA_2_ activity. To examine this, BBC was pre-incubated for 15 min with a cytoplasmic PLA_2_ antagonist documented to inhibit Golgi membrane tubules, ONO-RS-082 (de Figueiredo et al., 1998), prior to addition with Golgi pre-incubated with Gβ1γ2. The results showed that addition of Gβ1γ2 to low cytosol was able to stimulate Golgi membrane tubules, but Gβ1γ2 did not stimulate membrane tubules when cytosol was pretreated with ONO (Figure 5). These results indicate that Gβ1γ2 requires PLA_2_ activity found in BBC to stimulate Golgi membrane tubules.

## Discussion

Here we provide *in vitro* evidence that heterotrimeric G protein subunits Gβ1γ2 stimulate Golgi membrane tubule formation, which is dependent on PLA_2_ activity. Until now, the identified role of Gβ1γ2 at the Golgi complex has been limited to membrane fission in the generation of TGN vesicles. This work is consistent with the hypothesis that Gβγ subunits are additionally important for activation of PLA_2_ enzymes that stimulate membrane tubule formation.

Previous studies using the inhibitor ITD implicated Gβγ signaling in the regulation of PLA_2_ enzymes. ITD has been used to inhibit inflammatory signaling, by decreasing Gβγ activation of PLA_2_ enzymes (Hashizume et al., 1991). More recent studies show ITD inhibition of BFA-stimulated Golgi membrane tubule formation, suggesting a broader role of Gβγ regulation of PLA_2_ enzymes (Chan et al., 2004). Here we further explore the hypothesis that ITD inhibits Gβγ activation of PLA_2_ enzymes involved in the formation of Golgi membrane tubules. Using an *in vitro* reconstitution assay, we find that ITD inhibits BBC-stimulated membrane tubules from isolated Golgi complexes. This inhibition can be rescued by the addition of purified Gβγ subunits, further supporting the notion that ITD inhibits a Gβγ signaling pathway.

Heterotrimeric Gβγ subunits have recently been shown to localize to the Golgi complex upon GPCR stimulation (Saini et al., 2007; Irannejad and Wedegaertner, 2010; Saini et al., 2010). Once localized to the Golgi complex, Gβ1γ2 stimulates a signaling cascade that increases the TGN diacylglycerol levels necessary for the recruitment of PKD. Upon reaching the Golgi, PKD is subsequently activated by PKCη, leading to phosphorylation of PI4KIIIβand ceramide transfer protein (CERT), ultimately leading to enhanced fission and transport of cargo (Jamora et al., 1999; Baron and Malhotra, 2002; Hausser et al., 2005; Fugmann et al., 2007; Irannejad and Wedegaertner, 2010). Although this signaling cascade includes factors such as PKD, which have been implicated in vesicle fission (Liljedahl et al., 2001), the current model of cargo export from the Golgi complex does not address how the initial positive curvature to form vesicles and membrane tubules is generated to promote the increase in transport upon GPCR stimulation. Increased transport upon Gβγ signaling at the Golgi can be prevented by inactivation of PKD (Diaz Añel and Malhotra, 2005). This inhibition of PKD kinase activity results in the exacerbation of long and persistent TGN membrane tubules (Liljedahl et al., 2001). These results indicate that the machinery involved in the outward bending of Golgi membranes is activated, but the subsequent fission is prevented.

The TGN membrane tubules stimulated by PKD inactivation can be inhibited by the PLA_2_ inhibitor ONO (Schmidt et al., 2010). ONO prevents the formation of new PKD kinase dead (PKD-KD) TGN tubules, decreases the number of TGN tubules containing ts045 VSV-G, and inhibits the transport of ts045 VSV-G from the TGN to the plasma membrane. Consistent with this, the formation of Gβγ-stimulated tubules was sensitive to ONO inhibition when added to BBC. This suggests that PLA_2_ enzyme activity present in the cytosol is required for Gβ1γ2 stimulation of tubules, which are likely stimulated upstream of PKD. While it is possible that a separate signaling pathway regulates the formation of membrane tubules, it is conceivable that stimulation of both positive curvature for outward budding and negative curvature for fission are interconnected signaling pathways activated by Gβγ. The results presented here suggest the latter: Gβγ stimulates the outward curvature of Golgi membranes through PLA_2_ activation as well as PKD-dependent fission.

Specific PLA_2_ enzymes cPLA_2_α and PAFAH Ib are implicated in the formation of Golgi membrane tubules and TGN to plasma membrane transport (Regan-Klapisz et al., 2009; San Pietro et al., 2009; Bechler et al., 2010). cPLA_2_α has been shown to be important for inter-cisternal Golgi membrane tubule formation that aids in transport across the cisternal stack (San Pietro et al., 2009) and has been implicated in the transport of tight junction proteins to the plasma membrane (Regan-Klapisz et al., 2009). Additionally, PAFAH Ib has been shown to affect the localization of PKD to the TGN and VSV-G kinetics from the Golgi to the plasma membrane (Bechler et al., 2010). It will be interesting in the future to determine whether these specific PLA_2_ enzymes, or unidentified PLA enzymes, are part of the Gβγ signaling pathway at the Golgi and how they are interconnected with PKD-activated fission to generate transport carriers.

### Conflict of interest statement

The authors declare that the research was conducted in the absence of any commercial or financial relationships that could be construed as a potential conflict of interest.

## References

[B1] Akgoz M. Kalyanaraman V. Gautam N. (2006). G protein betagamma complex translocation from plasma membrane to Golgi complex is influenced by receptor gamma subunit interaction. Cell. Signal. 18, 1758–1768. 10.1016/j.cellsig.2006.01.016 16517125 PMC2230546

[B2] Akiba S. Kato E. Sato T. Fujii T. (1992). Biscoclaurine alkaloids inhibit receptor-mediated phospholipase A_2_ activation probably through uncoupling of a GTP-binding protein from the enzyme in rat peritoneal mast cells. Biochem. Pharmacol. 44, 45–50. 10.1016/0006-2952(92)90036-I 1632837

[B3] Akiba S. Nagatomo R. Ishimoto T. Sato T. (1995). Effect of berbamine on cytosolic phospholipase A_2_ activation in rabbit platelets. Eur. J. Pharmacol. 291, 343–350. 10.1016/0922-4106(95)90075-6 8719419

[B4] Banta M. Polizotto R. S. Wood S. A. De Figueiredo P. Brown W. J. (1995). Characterization of a cytosolic activity that induces the formation of Golgi membrane tubules in a cell-free reconstitution system. Biochemistry 34, 13359–13366. 10.1021/bi00041a012 7577921

[B5] Baron C. L. Malhotra V. (2002). Role of diacylglycerol in PKD recruitment to the TGN and protein transport to the plasma membrane. Science 295, 325–328. 10.1126/science.1066759 11729268

[B6] Bechler M. E. de Figueiredo P. Brown W. J. (2012). A PLA1-2 punch regulates the Golgi complex. Trends Cell Biol. 22, 116–124. 10.1016/j.tcb.2011.10.003 22130221 PMC3273632

[B7] Bechler M. E. Doody A. M. Racoosin E. Lin L. Lee K. H. Brown W. J. (2010). The phospholipase complex PAFAH Ib regulates the functional organization of the Golgi complex. J. Cell Biol. 190, 45–53. 10.1083/jcb.200908105 20624900 PMC2911670

[B8] Ben-Tekaya H. Kahn R. A. Hauri H. P. (2010). ADP ribosylation factors 1 and 4 and group VIA phospholipase A regulate morphology and intraorganellar traffic in the endoplasmic reticulum-Golgi intermediate compartment. Mol. Biol. Cell 21, 4130–4140. 10.1091/mbc.E10-01-0022 20881058 PMC2993742

[B9] Brown W. J. Chambers K. Doody A. (2003). Phospholipase A_2_ (PLA_2_) enzymes in membrane trafficking: mediators of membrane shape and function. Traffic 4, 214–221. 10.1034/j.1600-0854.2003.00078.x 12694560

[B10] Chan D. Strang M. Judson B. Brown W. J. (2004). Inhibition of membrane tubule formation and trafficking by isotetrandrine, an antagonist of G-protein-regulated phospholipase A_2_ enzymes. Mol. Biol. Cell 15, 1871–1880. 10.1091/mbc.E03-09-0644 14767064 PMC379283

[B11] Cluett E. B. Wood S. A. Banta M. Brown W. J. (1993). Tubulation of Golgi membranes *in vivo* and invitro in the absence of Brefeldin-A. J. Cell Biol. 120, 15–24. 10.1083/jcb.120.1.15 8416985 PMC2119494

[B12] de Figueiredo P. Drecktrah D. Katzenellenbogen J. A. Strang M. Brown W. J. (1998). Evidence that phospholipase A_2_ activity is required for Golgi complex and trans Golgi network membrane tubulation. Proc. Natl. Acad. Sci. U.S.A. 95, 8642–8647. 10.1073/pnas.95.15.8642 9671731 PMC21129

[B13] de Figueiredo P. Drecktrah D. Polizotto R. S. Cole N. B. Lippincott-Schwartz J. Brown W. J. (2000). Phospholipase A_2_ antagonists inhibit constitutive retrograde membrane traffic to the endoplasmic reticulum. Traffic 1, 504–511. 10.1034/j.1600-0854.2000.010608.x 11208136

[B14] de Figueiredo P. Polizotto R. S. Drecktrah D. Brown W. J. (1999). Membrane tubule-mediated reassembly and maintenance of the Golgi complex is disrupted by phospholipase A_2_ antagonists. Mol. Biol. Cell 10, 1763–1782. 10.1091/mbc.10.6.1763 10359595 PMC25369

[B15] Denker S. P. McCaffery J. M. Palade G. E. Insel P. A. Farquhar M. G. (1996). Differential distribution of α subunits and β γ subunits of heterotrimeric G proteins on Golgi membranes of the exocrine pancreas. J. Cell Biol. 133, 1027–1040. 10.1083/jcb.133.5.1027 8655576 PMC2120853

[B16] Diaz Añel A. M. Malhotra V. (2005). PKCη is required for β 1γ2/β 3γ2- and PKD-mediated transport to the cell surface and the organization of the Golgi apparatus. J. Cell Biol. 169, 83–91. 10.1083/jcb.200412089 15824133 PMC2171908

[B17] Drecktrah D. Brown W. J. (1999). Phospholipase A_2_ antagonists inhibit nocodazole-induced Golgi ministack formation: evidence of an ER intermediate and constitutive cycling. Mol. Biol. Cell 10, 4021–4032. 10.1091/mbc.10.12.4021 10588640 PMC25740

[B18] Fugmann T. Hausser A. Schoffler P. Schmid S. Pfizenmaier K. Olayioye M. A. (2007). Regulation of secretory transport by protein kinase D-mediated phosphorylation of the ceramide transfer protein. J. Cell Biol. 178, 15–22. 10.1083/jcb.200612017 17591919 PMC2064413

[B19] Ha K. D. Clarke B. A. Brown W. J. (2012). Regulation of the Golgi complex by phospholipid remodeling enzymes. Biochim. Biophys. Acta 1821, 1078–1088. 10.1016/j.bbalip.2012.04.004 22562055 PMC3399269

[B20] Hashizume T. Yamaguchi H. Sato T. Fujii T. (1991). Suppressive effect of biscoclaurine alkaloids on agonist-induced activation of phospholipase A_2_ in rabbit platelets. Biochem. Pharmacol. 41, 419–423. 10.1016/0006-2952(91)90539-H 1899790

[B21] Hausser A. Storz P. Martens S. Link G. Toker A. Pfizenmaier K. (2005). Protein kinase D regulates vesicular transport by phosphorylating and activating phosphatidylinositol-4 kinase IIIβ at the Golgi complex. Nat. Cell Biol. 7, 880–886. 10.1038/ncb1289 16100512 PMC1458033

[B22] Irannejad R. Wedegaertner P. B. (2010). Regulation of constitutive cargo transport from the *trans*-Golgi network to plasma membrane by Golgi-localized G protein betagamma subunits. J. Biol. Chem. 285, 32393–32404. 10.1074/jbc.M110.154963 20720014 PMC2952241

[B23] Jamora C. Takizawa P. A. Zaarour R. F. Denesvre C. Faulkner D. J. Malhotra V. (1997). Regulation of golgi structure through heterotrimeric G proteins. Cell 91, 617–626. 10.1016/S0092-8674(00)80449-3 9393855

[B24] Jamora C. Yamanouye N. Van Lint J. Laudenslager J. Vandenheede J. R. Faulkner D. J.. (1999). Gβ γ-mediated regulation of Golgi organization is through the direct activation of protein kinase D. Cell 98, 59–68. 10.1016/S0092-8674(00)80606-6 10412981

[B25] Liljedahl M. Maeda Y. Colanzi A. Ayala I. Van Lint J. Malhotra V. (2001). Protein kinase D regulates the fission of cell surface destined transport carriers from the *trans*-Golgi network. Cell 104, 409–420. 10.1016/S0092-8674(01)00228-8 11239398

[B26] Martinez-Alonso E. Tomas M. Martinez-Menarguez J. A. (2013). Golgi tubules: their structure, formation and role in intra-Golgi transport. Histochem. Cell Biol. 140, 327–339. 10.1007/s00418-013-1114-9 23812035

[B26a] Mayeenuddin L. H. McIntire W. E. Garrison J. C. (2006). Differential sensitivity of P-Rex-1 to isoforms of G protein betagamma dimers. J. Biol. Chem. 281, 1913–1920. 10.1074/jbc.M506034200 16301321

[B27] Polizotto R. S. De Figueiredo P. Brown W. J. (1999). Stimulation of Golgi membrane tubulation and retrograde trafficking to the ER by phospholipase A_2_ activating protein (PLAP) peptide. J. Cell. Biochem. 74, 670–683. 10.1002/(SICI)1097-4644(19990915)74:4<670::AID-JCB16>3.0.CO;2-# 10440936

[B28] Puertollano R. Aguilar R. C. Gorshkova I. Crouch R. J. Bonifacino J. S. (2001). Sorting of mannose 6-phosphate receptors mediated by the GGAs. Science 292, 1712–1716. 10.1126/science.1060750 11387475

[B29] Puertollano R. Van Der Wel N. N. Greene L. E. Eisenberg E. Peters P. J. Bonifacino J. S. (2003). Morphology and dynamics of clathrin/GGA1-coated carriers budding from the *trans*-Golgi network. Mol. Biol. Cell 14, 1545–1557. 10.1091/mbc.02-07-0109 12686608 PMC153121

[B30] Regan-Klapisz E. Krouwer V. Langelaar-Makkinje M. Nallan L. Gelb M. Gerritsen H.. (2009). Golgi-associated cPLA2α regulates endothelial cell-cell junction integrity by controlling the trafficking of transmembrane junction proteins. Mol. Biol. Cell 20, 4225–4234. 10.1091/mbc.E08-02-0210 19675210 PMC2754936

[B31] Saini D. K. Kalyanaraman V. Chisari M. Gautam N. (2007). A family of G protein β γ subunits translocate reversibly from the plasma membrane to endomembranes on receptor activation. J. Biol. Chem. 282, 24099–24108. 10.1074/jbc.M701191200 17581822 PMC2238721

[B32] Saini D. K. Karunarathne W. K. Angaswamy N. Saini D. Cho J. H. Kalyanaraman V.. (2010). Regulation of Golgi structure and secretion by receptor-induced G protein β γ complex translocation. Proc. Natl. Acad. Sci. U.S.A. 107, 11417–11422. 10.1073/pnas.1003042107 20534534 PMC2895111

[B33] San Pietro E. Capestrano M. Polishchuk E. V. Dipentima A. Trucco A. Zizza P.. (2009). Group IV phospholipase A_2_α controls the formation of inter-cisternal continuities involved in intra-Golgi transport. PLoS Biol. 7:e1000194. 10.1371/journal.pbio.1000194 19753100 PMC2732982

[B34] Schmidt J. A. Kalkofen D. N. Donovan K. W. Brown W. J. (2010). A role for phospholipase A_2_ activity in membrane tubule formation and TGN trafficking. Traffic 11, 1530–1536. 10.1111/j.1600-0854.2010.01115.x 20874826 PMC4906538

[B35] Stow J. L. Sabolic I. Brown D. (1991). Heterogeneous localization of G protein alpha-subunits in rat kidney. Am. J. Physiol. 261, F831–840. 1951714 10.1152/ajprenal.1991.261.5.F831

[B36] Takizawa P. A. Yucel J. K. Veit B. Faulkner D. J. Deerinck T. Soto G.. (1993). Complete vesiculation of Golgi membranes and inhibition of protein transport by a novel sea sponge metabolite, ilimaquinone. Cell 73, 1079–1090. 10.1016/0092-8674(93)90638-7 8513494

[B37] Waguri S. Dewitte F. Le Borgne R. Rouille Y. Uchiyama Y. Dubremetz J. F.. (2003). Visualization of TGN to endosome trafficking through fluorescently labeled MPR and AP-1 in living cells. Mol. Biol. Cell 14, 142–155. 10.1091/mbc.E02-06-0338 12529433 PMC140234

[B38] Yang J. S. Valente C. Polishchuk R. S. Turacchio G. Layre E. Moody D. B.. (2011). COPI acts in both vesicular and tubular transport. Nat. Cell Biol. 13, 996–1003. 10.1038/ncb2273 21725317 PMC3149785

